# Optimizing In-Hospital Mortality Prediction After Cardiac Surgery: A Machine Learning Approach Using Feature Engineering for Imbalanced Data

**DOI:** 10.3390/diagnostics16162557

**Published:** 2026-08-13

**Authors:** Po-Cheng Kao, Chih-Cheng Wu, Jung-Chun Yeh

**Affiliations:** 1 Department of Anesthesiology, Chung Shan Medical University Hospital, Taichung 40201, Taiwan; kaobocheng@gmail.com; 2 School of Medicine, Chung Shan Medical University, Taichung 40201, Taiwan; 3 Department of Anesthesiology, Taichung Veterans General Hospital, Taichung 40705, Taiwan; chihcheng.wu@gmail.com; 4 Department of Data Science and Big Data Analytics, Providence University, Taichung 43301, Taiwan; 5 Department of Financial Engineering, Providence University, Taichung 43301, Taiwan; 6 Department of Post-Baccalaureate Medicine, College of Medicine, National Chung Hsing University, Taichung 40227, Taiwan; 7 School of Dentistry, Chung Shan Medical University, Taichung 40201, Taiwan; 8 Department of Dentistry, Chung Shan Medical University Hospital, Taichung 40201, Taiwan

**Keywords:** cardiac surgery, in-hospital mortality, machine learning, feature engineering, SMOTE, explainable AI (XAI)

## Abstract

**Background/Objectives:** Cardiac surgery involves unique complexities that differ from those of general ICU populations. Traditional scoring systems often underperform due to the significant class imbalance between survival and mortality. This study utilized the MIMIC-IV database, integrating machine learning (ML) and feature engineering to develop an in-hospital mortality prediction model specifically for open-heart surgery patients. **Methods:** We included 6941 cases (mortality: 76, 1.095%). Sixty-eight variables from the first ICU day were extracted. Following data preprocessing and imputation, four ML models—logistic regression, random forest (RF), XGBoost, and multilayer perceptron (MLP)—were constructed using stratified 10-fold cross-validation. SMOTE was applied to address class imbalance. A streamlined 17-variable model was developed and compared against the Sequential Organ Failure Assessment (SOFA) and the Oxford Acute Severity of Illness Score (OASIS). **Results:** Among the 68-variable models, RF achieved the highest area under the receiver operating characteristic curve (AUROC) of 0.915 (95% CI, 0.855–0.966). For the 17-variable models, MLP performed best (AUROC: 0.920; 95% CI, 0.865–0.964), significantly outperforming SOFA (0.688) and OASIS (0.690). Regarding the area under the precision-recall curve (AUCPR), the 17-variable MLP also yielded the highest score (0.203; 95% CI, 0.060–0.389) compared with SOFA (0.161) and OASIS (0.046). SHapley Additive exPlanations (SHAP) analysis identified bicarbonate levels, mechanical ventilation, and mean pulmonary arterial pressure as the top predictors, consistent with clinical expectations. **Conclusions:** The streamlined MLP model significantly outperforms traditional scoring systems and may serve as a useful tool for early postoperative risk stratification after open-heart surgery.

## 1. Introduction

Compared with general surgical procedures, open-heart surgery is associated with a higher mortality rate due to its inherent complexity and the patients’ compromised physiological conditions. Accurate risk stratification, therefore, provides physicians with critical clinical guidance. The EuroSCORE II [1] and the Society of Thoracic Surgeons (STS) risk score [2,3] are commonly used for risk prediction in open-heart surgery. These assessments collect preoperative data to estimate the postoperative mortality rates and the risk of major complications. However, open-heart surgery often involves significant physiological shifts, such as those induced by cardiopulmonary bypass and substantial blood or fluid infusions, leading to dramatic changes in fluids and electrolytes. Additionally, the intricate surgical process is sensitive to intraoperative variables. Therefore, predicting postoperative risks based solely on preoperative information may be limited by unforeseen intraoperative events. Leveraging postoperative information can yield more accurate results, as it reflects the patient’s immediate clinical status following these events.

Despite its importance, there is still no universally recognized evaluation system for post-cardiac-surgery risks. Common risk assessment systems, such as the Simplified Acute Physiologic Score (SAPS II) [4], the Acute Physiology and Chronic Health Evaluation (APACHE II) [5], and the Sequential Organ Failure Assessment (SOFA) score [6], are widely used in intensive care units. However, due to the unique characteristics of open-heart surgery patients, these scoring systems were often designed with the exclusion of this group. Subsequent research has explored their applicability to these surgical patients, but they are mostly used as basic reference indicators [7,8]. Additionally, some studies have achieved promising results using specific parameters like vasopressor dosage [9] or blood lactate levels [10] to enhance predictions for these patients.

Machine learning has been widely used in the medical field in recent years [11,12]. By combining feature engineering methods, it is possible to select key features to provide a reference for clinical decision making. Previous studies have demonstrated the feasibility of machine learning for predicting postoperative mortality after cardiac surgery. However, many of these models were developed using a large number of predictors or primarily emphasized predictive performance, potentially limiting their clinical applicability and interpretability. Although reported in-hospital mortality rates after cardiac surgery range from approximately 1.9% to 3.4% [13,14,15], the marked imbalance between postoperative survival and mortality remains a significant methodological challenge [16]. This pronounced class imbalance highlights the importance of appropriate strategies for addressing imbalanced data in predictive model development. Therefore, rather than simply applying machine learning algorithms, this study aimed to develop a simplified and interpretable prediction model for postoperative open-heart surgery patients by integrating feature engineering, class imbalance handling, and SHAP-based model interpretation. We further compared its performance with conventional ICU scoring systems to evaluate its potential clinical applicability.

## 2. Materials and Methods

### 2.1. Data Source and Setting

Data were obtained from the Medical Information Mart for Intensive Care IV (MIMIC-IV) database [17]. The MIMIC-IV dataset comprises longitudinal health records from ICU patients at the Beth Israel Deaconess Medical Center (BIDMC) in Boston, MA, USA. It collects ICU hospitalization information recorded between 2008 and 2019, encompassing over 60,000 stays. The database contains de-identified patient demographics, medical records, diagnosis codes, vital signs, clinical procedures, laboratory data, medication therapy, and intensive care records. Access to the database was granted following the completion of the Collaborative Institutional Training Initiative (CITI) program (certification number: 39830591).

Data mining was conducted from March to August 2025. The pgAdmin 4 (version 5.2; The pgAdmin Development Team, PostgreSQL Global Development Group) interface was utilized to extract and organize data from MIMIC-IV using PostgreSQL (version 13; PostgreSQL Global Development Group, Berkeley, CA, USA). Data analysis and model development were performed using Python (version 3.11; Python Software Foundation, Wilmington, DE, USA) on the Google Colaboratory platform (Google LLC, Mountain View, CA, USA).

### 2.2. Patient Selection and Outcome Measures

The study cohort consisted of patients undergoing isolated open-heart surgery. The inclusion criteria were age ≥18 years and admission to the ICU within 24 h postoperatively with an ICU stay exceeding 24 h. Patients undergoing heart transplantation, simultaneous major vascular surgery (e.g., aortic surgery), or transcatheter valvular interventions were excluded. For patients with multiple ICU admissions during the same hospitalization, data from the initial surgery associated with the index ICU admission were selected for analysis. The International Classification of Diseases, Ninth and Tenth Revisions (ICD-9 and ICD-10) codes within the MIMIC-IV database were utilized to identify patients undergoing coronary artery bypass grafting (CABG), as well as repair or replacement of the aortic, mitral, tricuspid, or pulmonary valves.

The primary objective of this study was to develop and evaluate machine learning predictive models for in-hospital mortality. The secondary objective involved interpreting the optimal model to identify potential clinical predictors associated with mortality.

### 2.3. Parameters

This study extracted parameters incorporated in commonly used ICU scoring systems alongside clinical indicators used to monitor patients following cardiac surgery. For hemodynamic monitoring data, such as blood pressure and heart rate, the maximum, minimum, and average values recorded within the first 24 h of ICU admission were calculated. Regarding laboratory data, such as electrolytes and blood glucose levels, the maximum and minimum values obtained during the first day were included.

Initially, 132 candidate parameters were identified. Variables with missing data exceeding 50% were excluded because such variables were unlikely to represent routinely collected postoperative monitoring parameters in clinical practice. Since the objective of this study was to develop a prediction model based on commonly available postoperative ICU variables, retaining variables with extensive missingness was considered less clinically relevant and could introduce substantial uncertainty during data imputation. Following commonly adopted recommendations [18], variables with |r| > 0.7 were considered highly correlated, and one of the correlated variables was removed to reduce multicollinearity and enhance model interpretability. Additionally, since these patients often require inotropic and vasopressor support postoperatively, the Vasoactive-Inotropic Score (VIS) [19] was employed to quantify and integrate pharmacological support levels. The maximum VIS calculated on the first day of ICU admission has been shown to be a potent predictor of patient prognosis [9]. Following these selection and integration processes, a final set of 68 parameters was included in the analysis.

### 2.4. Data Preprocessing

The cohort was randomly partitioned into training and validation sets at an 8:2 ratio. To preserve the original class distribution, we utilized stratified sampling to ensure that the proportions of survival and mortality remained consistent across both subsets. The independent validation dataset was held out before any preprocessing, feature selection, hyperparameter optimization, model training, or cross-validation and was used exclusively for final model evaluation.

MIMIC-IV, an electronic health record (EHR) system, utilizes automated processes to record clinical parameters. During the measurement process, interference may occur; while manual records allow for removal or remeasurement, electronic records may directly document such errors without adjustment. Additionally, certain values requiring manual input may contain issues such as input errors or discrepancies in measurement units. Therefore, all continuous physiological and laboratory variables were independently reviewed by an experienced physician using predefined physiological limits. Values beyond these limits were considered implausible and were treated as missing values before imputation.

Outliers are traditionally defined as values falling outside 1.5 times the interquartile range (IQR). However, as extreme physiological values may still exist in clinical practice, this study removed only extreme outliers—defined as values exceeding 3 times the IQR [20]. This approach aimed to minimize the impact of collection errors while retaining meaningful clinical variability.

Regarding missing data, simple imputation methods (e.g., mean or median) can lead to information loss, reduced variability, weakened impact of observed values, and the disregard of data relationships [21]. In this study, Multiple Imputation by Chained Equations (MICE) was employed to impute missing values [22]. MICE assumes that missing values are Missing at Random (MAR) and are generated from observed data. Imputation was implemented using the miceforest package (version 5.6.3; PyPI, USA) [23], which employs LightGBM as the default prediction model for chained imputation. Four imputed datasets were generated, with each dataset undergoing three MICE iterations before completion. These datasets were statistically combined to yield a single, complete dataset for analysis. Missing-value imputation was performed independently on the training and validation datasets to prevent information leakage between data partitions.

Categorical variables were transformed using frequency encoding, where each category was represented by its relative frequency in the training dataset. Frequency encoding was selected because it provides a compact numerical representation of categorical features without increasing feature dimensionality, as occurs with one-hot encoding [24]. Unlike target encoding, it does not incorporate outcome information during feature transformation, thereby avoiding the risk of target leakage. The numerical features were normalized via min–max scaling to a range of 0 to 1.

To address the significant class imbalance between survival and mortality, which often biases classifiers toward the majority class, several approaches have been proposed [25,26], including random undersampling, random oversampling, class weighting, cost-sensitive learning, Adaptive Synthetic Sampling (ADASYN), and the Synthetic Minority Oversampling Technique (SMOTE). Among these methods, SMOTE is one of the most widely adopted approaches in medical machine learning because it generates synthetic minority samples rather than simply duplicating existing observations, thereby reducing the risk of overfitting while preserving the underlying characteristics of the minority class [27]. Considering the extremely low in-hospital mortality rate in our cohort, SMOTE was selected as an appropriate and well-validated method for handling severe class imbalance. For instance, in a dataset with a 1% mortality rate, a classifier could achieve 99% accuracy by predicting all patients as survivors. SMOTE was then applied to the training dataset to generate synthetic minority samples and mitigate the effect of class imbalance during model development. Specifically, during each iteration of the stratified 10-fold cross-validation, the training subset was first identified, after which SMOTE was applied only to that training subset before model fitting. The corresponding validation subset retained its original class distribution and was never subjected to oversampling. This procedure minimized the risk of data leakage and ensured a realistic evaluation of model performance.

### 2.5. Model Development and Comparison

We utilized four machine learning algorithms for prediction: logistic regression (LR), random forest (RF), multilayer perceptron (MLP), and eXtreme Gradient Boosting (XGBoost). During the modeling process, we stratified the training dataset into 10 equal subsets based on the original class proportions. For each iteration, one fold was used as the testing set, while the remaining folds served as the training set—an approach referred to as stratified 10-fold cross-validation. Cross-validation prevented the model from depending exclusively on a specific training and testing dataset, thereby reducing bias. It also enabled the selection of the optimal model for further performance evaluation on the validation dataset [28].

To optimize each model, hyperparameter tuning was performed using the random search method with 10-fold stratified cross-validation [29]. This method involved randomly selecting parameter combinations and performing cross-validation to evaluate model performance. Random search is generally more efficient than grid search and is particularly suitable for cases with a broad parameter space or limited computational resources. The optimal hyperparameters were selected based on the mean recall score, and the predefined search spaces and optimization settings are provided in Supplementary Table S1.

The four machine learning models employed in this study are capable of ranking variable importance. Common thresholds for variable selection reported in previous studies include 10%, 25%, 50%, and log_2_*N* [30]. After identifying the optimal model through hyperparameter optimization and stratified 10-fold cross-validation on the training dataset, feature importance was calculated once using the entire training dataset. The independent validation dataset was not involved in feature selection or any stage of model development. Accordingly, these candidate thresholds were evaluated using this optimal model. Among them, selecting the top 25% of variables achieved the best predictive performance while maintaining model simplicity and was therefore adopted in the subsequent analyses. The reduced-feature models were subsequently developed using the same model development pipeline and evaluated using the independent validation dataset. A detailed analytical workflow of data preprocessing, feature selection, model development, and independent validation is provided in Supplementary Figure S1.

Finally, we used the validation dataset to compare the predictive performance among all variables, the subset of variables identified through feature selection, and traditional ICU scoring systems. Traditional ICU scoring systems frequently serve as benchmarks for evaluating new models [31]. In this study, we adopted the Sequential Organ Failure Assessment (SOFA) and the Oxford Acute Severity of Illness Score (OASIS) [32] for comparison. Although the APACHE II score is widely used in intensive care settings, certain necessary variables are unavailable in the MIMIC-IV dataset. Thus, OASIS was chosen as a comparative scoring system for this study. Previous research has also supported the use of OASIS as an alternative when the APACHE II score is not feasible [32].

### 2.6. Statistical Analysis and Model Interpretation

We evaluated multiple metrics to assess the models’ discriminative ability, including accuracy, precision, recall, F1-score, and area under the receiver operating characteristic (ROC) curve (AUROC). Model calibration was additionally evaluated using calibration curves and the Brier score based on the predicted probabilities generated by the final models on the independent validation dataset. Due to the inherent class imbalance, we placed greater emphasis on two indicators: recall, which reflects the model’s performance on positive samples, and the F1-score, which represents the harmonic mean of precision and recall. While the AUROC is a widely used metric for evaluating classification models, the significant prevalence of negative samples in this study could lead to a disproportionately low false positive rate (1 − specificity). Consequently, the model evaluation may become biased toward negative samples, potentially masking its performance on positive samples. To address this, we also employed the precision-recall curve, as precision and recall calculations do not incorporate true negatives, focusing solely on the model’s ability to correctly identify positive samples. In imbalanced datasets, the precision-recall curve and its area under the curve (AUCPR) may provide a more robust reflection of model performance than the ROC curve [33]. All evaluation metrics were reported as mean estimates with 95% confidence intervals, obtained through 2000 bootstrap resampling iterations on the validation dataset.

Model interpretation is crucial in medical applications, as it helps improve model transparency, supports clinical decision making, and allows experts to assess model reliability while identifying potential errors in data collection. This study employed Shapley Additive Explanations (SHAP) for model interpretation (version 0.51.0) [34]. SHAP is based on Shapley values from cooperative game theory, which quantify the contribution of individual features to the model’s prediction for a given observation. This ensures consistency and fairness in the interpretation process. By utilizing SHAP, we were able to elucidate the feature contributions of the model, quantify the contribution of each feature to the model predictions, and ultimately enhance model transparency and interpretability.

## 3. Results

**Figure 1:**
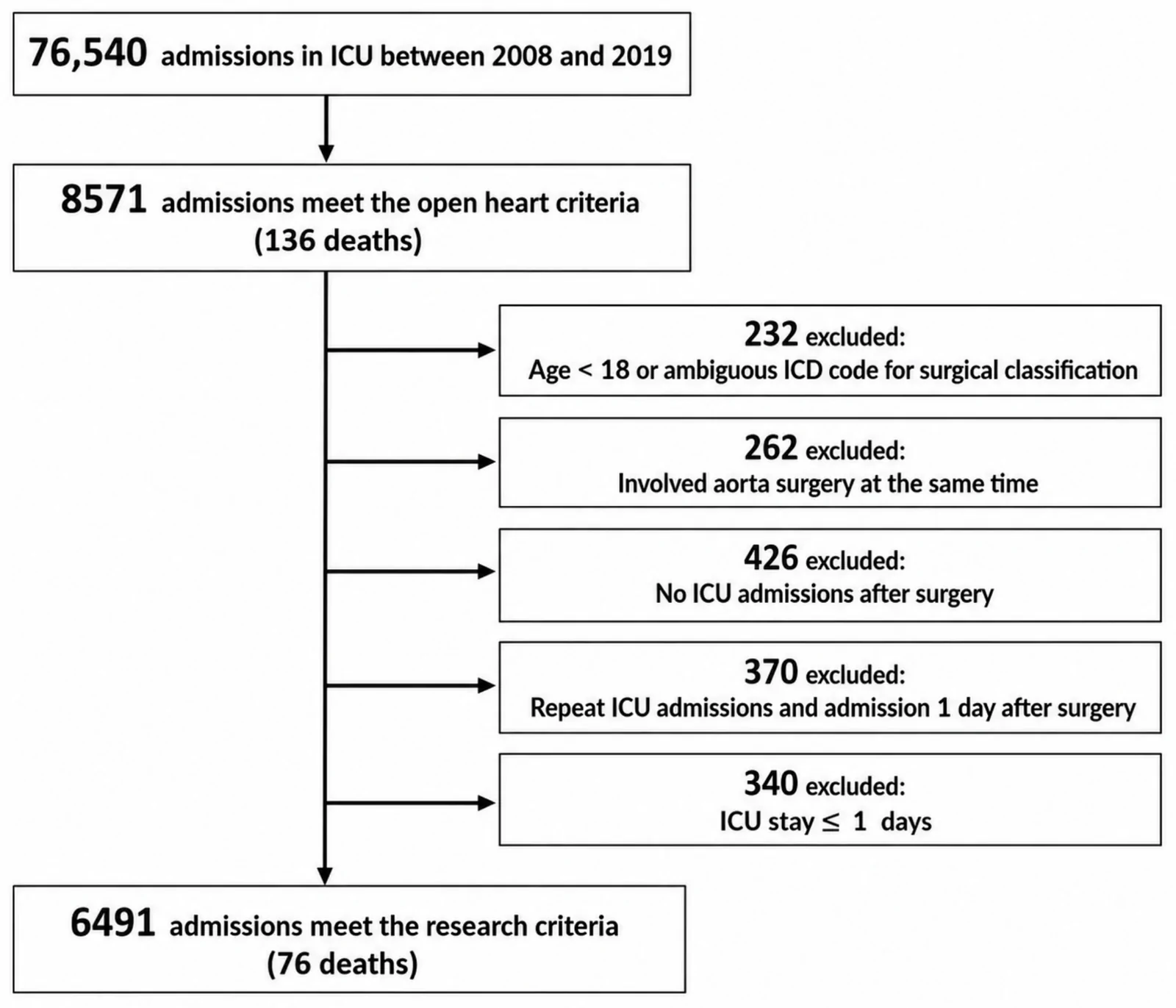
Patient recruitment and enrollment process.

The patient enrollment process is illustrated in Figure 1. A total of 6941 ICU admissions (representing 6906 patients) were included in the analysis, among which 76 deaths occurred during hospitalization (1.095%). Following random allocation, the training set comprised 5552 admissions with 61 deaths (1.099%), while the validation set contained 1389 admissions with 15 deaths (1.080%). The distribution of cardiac surgery types is detailed in Supplementary Table S2, with coronary artery bypass grafting identified as the most common procedure (54.3%), followed by aortic valve-related procedures (17.7%).

Regarding data completeness, most patients had fewer than 10 missing variables; specifically, 5 patients had more than 30 missing values, and 3 had more than 40. Details concerning missing data patterns are presented in Supplementary Figures S2 and S3. Descriptive statistics for all variables are provided in Supplementary Table S3.

Based on the performance of the four machine learning models on the training set (see Supplementary Table S4), the RF model demonstrated superior predictive performance. Therefore, the RF model was utilized to rank variable importance, leading to the selection of the top 25% of variables (*n* = 17) for subsequent analysis.

### 3.1. Performance Comparison of Models

Model performance was evaluated using both the full set of 68 variables and the subset of 17 selected variables. The predictive results for both sets are summarized in Table 1, and the corresponding hyperparameters are listed in Supplementary Table S5. When all 68 variables were incorporated, the RF model achieved the highest AUROC at 0.915 (95% CI, 0.855–0.966). In contrast, under the feature-reduced condition (17 variables), the MLP performed best with an AUROC of 0.920 (95% CI, 0.865–0.964).

Regarding the F1-score, the MLP yielded the highest score among the 68-variable models at 0.115 (95% CI, 0.040–0.200), with the RF showing comparable performance. When utilizing 17 variables, the MLP again demonstrated the best performance with an F1-score of 0.110 (95% CI, 0.053–0.169), while the other models showed consistent results.

In terms of recall, XGBoost achieved the highest value in both analyses—0.795 (95% CI, 0.556–1.000) for both the 68-variable and 17-variable sets. Overall, performance differences among the machine learning algorithms were smaller when using the 17 selected variables than when using all 68 variables.

For the validation dataset, the results and confusion matrices of models trained without and with SMOTE are presented in Supplementary Table S6. Both models were evaluated using the same independent validation dataset; the only difference was whether SMOTE was applied during model training. Models trained with SMOTE consistently achieved higher recall across all machine learning algorithms.

Calibration analysis was additionally performed on the independent validation dataset. Calibration curves and Brier scores are presented in Supplementary Figure S4. The RF and streamlined MLP model yielded Brier scores of 0.145 and 0.096, respectively.

### 3.2. Comparison with Traditional ICU Scoring Systems

Using the same validation dataset, the SOFA and OASIS scoring systems were evaluated and compared against the machine learning models that achieved the best AUROC under both 68-variable and 17-variable conditions (Table 2). The AUROC values for SOFA and OASIS were 0.688 (95% CI, 0.531–0.836) and 0.690 (95% CI, 0.564–0.814), respectively. Notably, both systems yielded a recall of 0, indicating that no in-hospital mortality cases were correctly identified. Furthermore, their AUROCs were significantly lower than those of the machine learning models (*p* < 0.05). Additionally, both systems demonstrated an accuracy of 0.989 (95% CI, 0.983–0.994). As this value closely approximates the proportion of negative cases, the results indicate that nearly all patients were classified as negative by both scoring systems.

**Figure 2:**
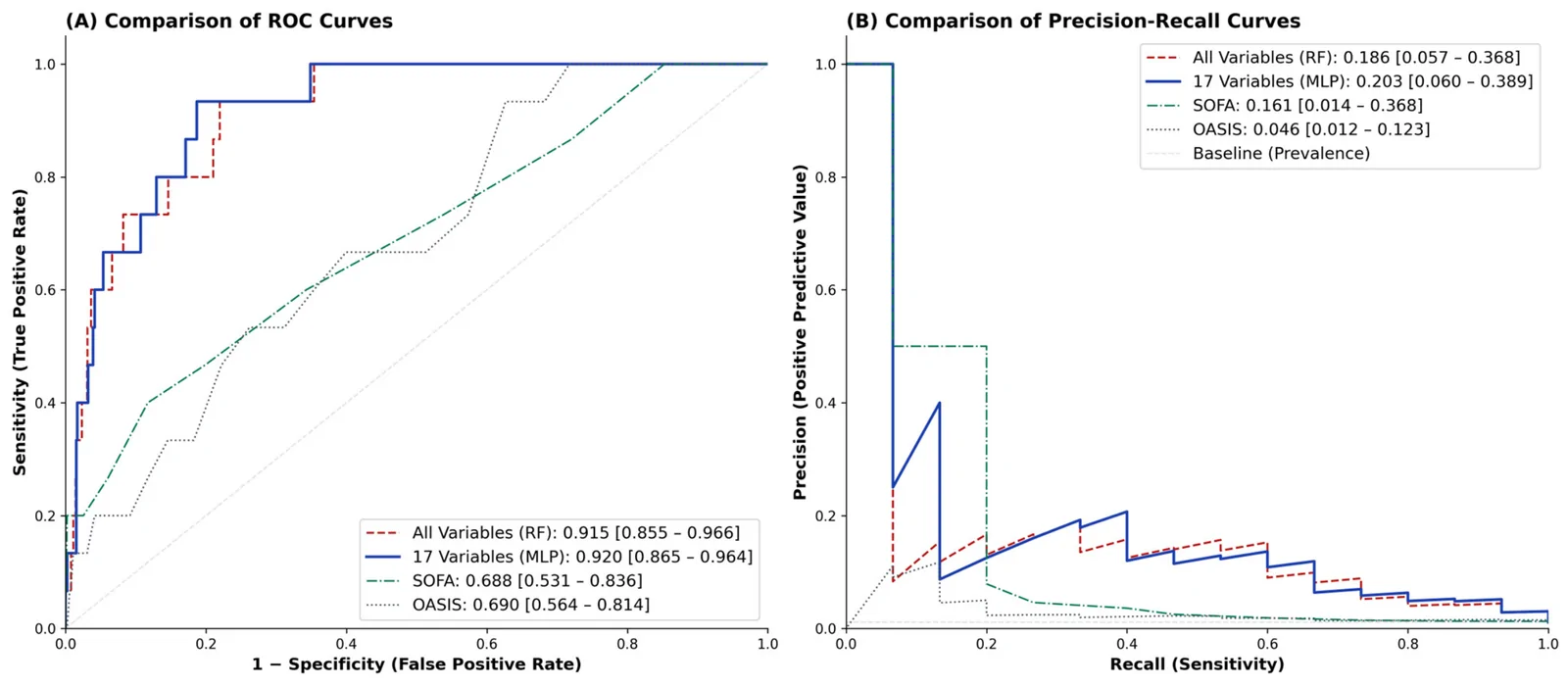
**Comparison of Predictive Models against ICU Scoring Systems.** (**A**) ROC curves and (**B**) Precision-Recall curves for mortality prediction. The machine learning models, particularly the 17-variable MLP, significantly outperformed the SOFA and OASIS in both discriminative power (AUROC) and positive class identification (AUCPR) under class imbalance. Abbreviations: RF, Random Forest; MLP, Multi-Layer Perceptron; SOFA, Sequential Organ Failure Assessment; OASIS, Oxford Acute Severity of Illness Score.

The comparative performance in terms of AUROC and AUCPR is illustrated in Figure 2. The AUROC values of all machine learning models outperformed those of the ICU scoring systems, with the 17-variable MLP achieving the best performance at 0.920 (95% CI, 0.865–0.964). The AUCPR results also demonstrated the superiority of machine learning models, where the 17-variable MLP attained the highest value at 0.203 (95% CI, 0.060–0.389). Both machine learning models significantly surpassed the AUCPR baseline (approximately 0.011). The two line plots in Figure 2 further illustrate that, for a given recall level (e.g., 0.7), the machine learning models consistently achieved lower false positive rates and higher precision than the traditional ICU scoring systems.

### 3.3. Model Interpretation

**Figure 3:**
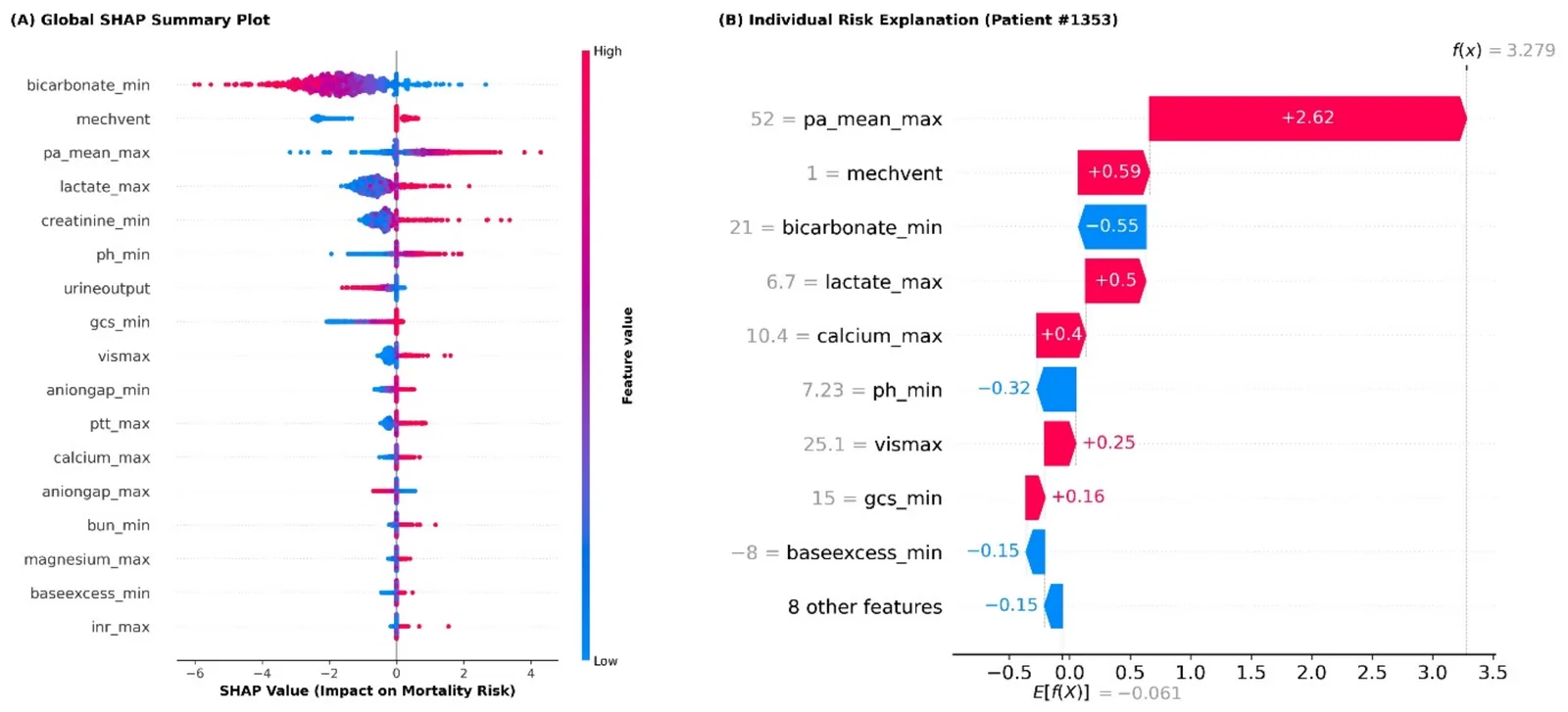
SHAP Interpretation of the final 17-Variable MLP Model. (**A**) Global Summary Plot: Displays the global impact of features on model output. Features are ranked by importance on the Y-axis. The X-axis (SHAP value) indicates the direction and magnitude of each feature’s contribution: positive values (right) increase the predicted risk, while negative values (left) decrease it. Each point represents a patient, with colors indicating feature values (red: high; blue: low). (**B**) Individual SHAP waterfall plot for a representative high-risk patient (#1353). The model predicted a high mortality risk (94.4% probability of death), primarily driven by elevated pa_mean_max (52 mmHg) and mechanical ventilation (mechvent = 1).

The 17-variable MLP model was selected for interpretation via SHAP, as it demonstrated superior performance across most evaluation metrics (AUROC, AUCPR, etc.). The SHAP summary plot (Figure 3A) illustrates feature importance and its impact on model output, with minimum bicarbonate levels, ventilator use, and mean pulmonary artery pressure identified as the top three contributing features. The influence of most variables aligned with clinical expectations; for instance, lower bicarbonate values (bicarbonate_min), higher creatinine levels (creatinine_min), elevated lactate levels (lactate_max), and increased vasopressor support (vismax) contributed to higher predicted mortality risk. However, the contribution patterns of pH and the GCS score deviated from conventional clinical expectations, warranting further investigation. Beyond population-level analysis, SHAP facilitated the interpretation of individualized patient predictions. Figure 3B illustrates the local explanation for one representative high-risk patient (#1353), who was predicted to have a 94.4% probability of death. The high predicted risk was primarily attributable to the contributions of elevated pulmonary artery pressure, ventilator dependence on the first postoperative day, and elevated lactate levels. Additional representative SHAP waterfall plots for intermediate- and low-risk patients are provided in Supplementary Figure S5, illustrating the feature contributions for patients across different predicted risk levels.

## 4. Discussion

The results of this study demonstrate that machine learning-based models significantly outperform traditional ICU scoring systems in predicting in-hospital mortality among patients undergoing cardiac surgery. Rather than simply demonstrating the superiority of machine learning algorithms, this study further aimed to develop a clinically applicable prediction framework by integrating feature engineering, class imbalance handling, and model interpretability. This superiority is particularly evident in model recall, which is critical for identifying high-risk patients in imbalanced clinical datasets. Our findings indicate that tree-based models, such as RF and XGBoost, initially exhibited superior predictive performance compared with the LR and MLP models prior to feature selection. However, following rigorous feature selection, the performance disparities among these models were substantially reduced. Similar findings have been reported by Christodoulou et al., who compared machine learning methods with logistic regression in clinical prediction models [35]. With appropriate data preprocessing and feature selection, predictive performance can be optimized and stabilized across different algorithmic architectures [35,36]. Consequently, a streamlined model—specifically the 17-variable MLP—achieved predictive performance comparable to the RF model utilizing the full feature set. The comparable performance achieved by the reduced-feature model suggests that a considerable proportion of the predictive information contained in the original feature set was redundant. Many variables reflected overlapping physiological processes, such as acid–base balance, tissue perfusion, renal function, and cardiopulmonary status. Therefore, eliminating redundant or less informative variables did not substantially compromise predictive performance and may even reduce model noise, thereby improving generalizability. Maintaining comparable predictive performance with substantially fewer variables may facilitate future implementation in routine postoperative cardiac intensive care, where model simplicity and interpretability are essential for clinical decision support.

In our study, the in-hospital mortality rate after cardiac surgery was 1.095%, which is slightly lower than the values reported in previous studies. This difference may be attributable to the study population’s selection criteria, including the inclusion of only patients admitted to the ICU postoperatively, the exclusion of emergency surgical cases, and the restriction of the analysis to patients with an ICU stay longer than 24 h. Consequently, patients who died preoperatively, intraoperatively, or within the first postoperative day were excluded. Nevertheless, the impact of data imbalance on model performance still warrants careful consideration.

Accuracy is commonly used to evaluate predictive models; however, in this study, positive cases were far fewer than negative cases. In such imbalanced settings, models that classify all patients as survivors can still yield high accuracy, a phenomenon known as the “accuracy paradox” [37,38], which was clearly observed in traditional ICU scoring systems in this study. As the primary objective of our research was to identify patients at risk of in-hospital mortality, recall was considered a more clinically meaningful metric. Consistent with this objective, the machine learning models maintained lower false positive rates and higher precision than the traditional ICU scoring systems across comparable recall levels, further supporting their clinical utility in identifying high-risk patients. Additionally, following the application of SMOTE, the recall improved across all models, affirming that SMOTE is effective in addressing class imbalance [16,26,27]. Cross-validation was subsequently employed to mitigate the risk of overfitting. In addition, the AUROC and AUCPR were used for comprehensive model evaluation in this imbalanced clinical dataset [16,27]. These metrics consistently suggested that machine learning-based models provide more favorable predictive performance than traditional approaches. Considering the extremely low mortality rate in our cohort, these findings also demonstrate the importance of incorporating imbalance-handling strategies when developing prediction models for postoperative cardiac surgery patients.

Calibration was additionally evaluated using calibration curves and the Brier score to complement the discrimination metrics. Although the proposed models demonstrated excellent discrimination, their calibration performance was comparatively less optimal. This may partially be attributable to the use of SMOTE to address the severe class imbalance during model development, as oversampling methods may alter the estimated class probabilities. Given that the primary objective of this study was to develop models with high discrimination for identifying patients at risk of in-hospital mortality after cardiac surgery, the additional calibration assessment provides complementary information without altering the overall interpretation of model performance.

Model interpretability is indispensable in medical applications. It facilitates understanding of model behavior, provides insights into how individual features contribute to model predictions, and supports clinical decision making by mitigating reliance on opaque “black-box” model outputs [39]. Since errors inevitably arise during data collection, the integration of model interpretation with domain expertise serves as a critical mechanism to assess model reliability. SHAP is a model-agnostic post hoc interpretability method that effectively elucidates the behavior of complex models, thereby enhancing transparency and supporting trustworthy medical decision making [39].

The SHAP analysis showed that the feature contribution patterns identified by the model were largely consistent with clinical expectations and the existing literature. Bicarbonate_min was identified as the most influential feature, highlighting the critical role of acid–base balance in patient prognosis. Consistently, elevated postoperative pulmonary artery pressure (pa_mean_max) has been reported to be associated with adverse complications and mortality following cardiac surgery [40,41,42]. Furthermore, elevations in lactate_max and creatinine_min, alongside the requirement for mechanical ventilation (mechvent), were identified as important features contributing to the model’s mortality predictions. These factors reflect the profound impact of systemic tissue perfusion, renal function, and respiratory failure on clinical outcomes [10,43,44,45]. In summary, the systemic inflammatory response induced by cardiopulmonary bypass, coupled with potential postoperative low cardiac output syndrome, can severely disrupt metabolic homeostasis. This often manifests as electrolyte imbalances and acid–base abnormalities; if not promptly addressed, these derangements significantly increase the risk of mortality and multisystem complications.

In this study, we also observed two findings that initially deviated from clinical expectations. First, a higher minimum pH value within the first 24 h (pH_min) was associated with a higher predicted mortality risk in our model. Although clinical focus typically centers on acidosis, our results indicate that alkalosis is also a significant predictor of mortality in critically ill patients, aligning with previous research [46,47]. Pathophysiologically, an elevated pH shifts the oxygen–hemoglobin dissociation curve to the left, increasing hemoglobin–oxygen affinity and potentially exacerbating tissue hypoxia. Moreover, iatrogenic factors—such as excessive bicarbonate administration for acidosis correction or ventilator-induced hyperventilation—may lead to metabolic or respiratory alkalosis, reflecting either impaired oxygen delivery or overtreatment. Therefore, the mortality risk associated with higher pH levels is likely multifactorial [46], necessitating further investigation to elucidate the exact underlying mechanisms. Second, regarding the GCS component (gcs_min), higher scores appeared to be associated with an increased mortality risk. This paradox is likely attributable to the specific data-coding mechanism in MIMIC-IV. According to the official documentation, critically ill patients who are intubated or sedated are often assigned a GCS of 15 to reflect their baseline neurological status under these conditions. Consequently, high scores captured by the model may inadvertently serve as a proxy for severely ill patients requiring sedation or mechanical ventilation. While this data characteristic potentially confounds the model’s interpretation, GCS remains a pivotal monitoring parameter in the ICU; thus, we elected to retain it in our model. Future studies should consider evaluating the Eye, Verbal, and Motor components separately to attain more granular prognostic results.

Our findings demonstrate that machine learning can be used to predict in-hospital mortality after cardiac surgery, achieving good performance across different models. After feature selection, similar predictive results can be obtained using simpler models. Feature selection reduces computational cost, accelerates training, improves performance, mitigates overfitting, and enhances model interpretability. Overall, our study shows that with appropriate feature engineering and the handling of imbalanced data, machine learning models can outperform traditional ICU prediction metrics. This highlights another key conclusion: proper data preprocessing is as important as the choice of a predictive model.

### Limitations

This study has several limitations. First, the data were obtained from a single hospital, with a predominantly White patient population (74.5%). Although an independent validation cohort from the same database was used, external validation in other institutions and more diverse patient populations was not performed. Therefore, the generalizability of the proposed models across different institutions, patient populations, and perioperative management strategies remains uncertain. Second, although stratified cross-validation, SMOTE within the training folds, and evaluation using an independent validation cohort were employed to improve model robustness, the relatively small number of in-hospital deaths (*n* = 76) may still increase the risk of model instability and optimistic performance estimates. Future studies with larger cohorts and more outcome events are warranted to further validate the proposed models. Third, the raw data contained a substantial number of erroneous values (e.g., glucose values of 99,999). During data cleaning, extreme outliers exceeding three times the interquartile range (3 × IQR) were removed and imputed to reduce potential bias; however, some abnormal values may occur in real clinical settings, and their removal could result in the loss of important clinical information. In addition, the sampling frequency varied considerably across variables. Vital signs were recorded frequently, whereas laboratory measurements were relatively sparse, sometimes limited to one or two values per day. Modeling variables using only the maximum, minimum, or mean values may introduce bias in variable associations and fail to capture temporal trends, thereby affecting model predictions. Finally, although SHAP analysis provides insights into the contribution of individual features to model predictions, clinical outcomes are determined by the complex interactions among multiple variables rather than any single predictor alone.

## 5. Conclusions

Cardiac surgery requires intensive postoperative monitoring. Using commonly recorded ICU parameters, our machine learning models outperformed traditional scoring systems in predicting in-hospital mortality for rare clinical events. These findings suggest that machine learning models may provide a useful approach for early postoperative risk stratification using routinely available ICU data. Nevertheless, prospective external validation across different institutions, patient populations, and perioperative management strategies is required before routine clinical implementation.

## Figures and Tables

**Table 1:** Model performance across different variable sets.

Variable Set	Model	Accuracy	Precision	Recall	F1-Score	AUROC
68 Variables	LR	0.837 [0.816–0.856]	0.035 [0.013–0.061]	0.527 [0.267–0.786]	0.065 [0.025–0.112]	0.807 [0.693–0.911]
	RF	0.875 [0.857–0.892]	0.061 [0.028–0.097]	0.729 [0.467–0.944]	0.112 [0.054–0.174]	**0.915** **[0.855–0.966]**
	XGBoost	0.838 [0.818–0.857]	0.051 [0.025–0.083]	**0.795** **[0.556–1.0]**	0.096 [0.047–0.149]	0.897 [0.807–0.963]
	MLP	**0.924** **[0.910–0.937]**	**0.066** **[0.022–0.119]**	0.459 [0.200–0.722]	**0.115** **[0.040–0.200]**	0.807 [0.684–0.907]
17 variables	LR	0.847 [0.828–0.865]	0.054 [0.026–0.085]	0.794 [0.556–1.0]	0.101 [0.050–0.154]	0.898 [0.823–0.959]
	RF	**0.863** **[0.844–0.880]**	0.055 [0.026–0.089]	0.729 [0.467–0.944]	0.103 [0.049–0.160]	0.903 [0.822–0.965]
	XGBoost	0.850 [0.830–0.870]	0.055 [0.027–0.087]	**0.795** **[0.556–1.000]**	0.103 [0.052–0.159]	0.900 [0.819–0.963]
	MLP	0.862 [0.844–0.880]	**0.060** **[0.028–0.094]**	0.794 [0.556–1.0]	**0.110** **[0.053–0.169]**	**0.920** **[0.865–0.964]**

Note: Bold values indicate the best performance for each evaluation metric within each feature set. Numbers outside the brackets represent the mean metric value. Numbers inside the brackets (e.g., [0.816–0.856]) represent the 95% confidence interval (95% CI) of the metric. Abbreviations: LR, Logistic Regression; RF, Random Forest; XGBoost, eXtreme Gradient Boosting; MLP, Multi-Layer Perceptron; AUROC, Area Under the Receiver Operating Characteristic curve.

**Table 2:** Predictive performance comparison between the optimized machine learning models and traditional ICU scoring systems.

Model	Accuracy	Precision	Recall	F1-Score	AUROC	*p*-Value *
RF	0.875 [0.857–0.892]	**0.061** **[0.028–0.097]**	0.729 [0.467–0.944]	**0.112** **[0.054–0.174]**	0.915 [0.855–0.966]	-
MLP17	0.862 [0.844–0.880]	0.060 [0.028–0.094]	**0.794** **[0.556–1.0]**	0.110 [0.053–0.169]	**0.920** **[0.865–0.964]**	0.800
SOFA	**0.989** **[0.983–0.994]**	0 [0.000–0.000]	0 [0.000–0.000]	0 [0.000–0.000]	0.688 [0.531–0.836]	<0.001
OASIS	**0.989** **[0.983–0.994]**	0 [0.000–0.000]	0 [0.000–0.000]	0 [0.000–0.000]	0.690 [0.564–0.814]	0.002

Notes: Bold values indicate the best performance for each evaluation metric within each feature set. Numbers outside the brackets represent the mean metric value. Numbers in brackets (e.g., [0.857–0.892]) represent the 95% confidence interval (95% CI) of the metric. * *p*-value: Calculated by comparing the AUROC of the corresponding model/system against the RF model using the DeLong method. Abbreviations: RF, Random Forest using all variables; MLP17, Multi-Layer Perceptron using 17 variables; SOFA, Sequential Organ Failure Assessment; OASIS, Oxford Acute Severity of Illness Score; AUROC, Area Under the Receiver Operating Characteristic curve.

## Data Availability

The data used in this study are available from the Medical Information Mart for Intensive Care IV (MIMIC-IV) database (https://physionet.org/content/mimiciv/ (accessed on 30 June 2026)). Access is restricted to registered users who have completed the required ethics training. Further details regarding the dataset and methodology are available from the corresponding author upon reasonable request.

## Supplementary Materials

The following supporting information can be downloaded at: https://www.mdpi.com/article/10.3390/diagnostics16162557/s1, Table S1: Hyperparameter optimization settings and search spaces. Table S2: Distribution of cardiac surgery types by number of cases. Table S3: Descriptive statistics for all variables. Table S4: Model performance on the training dataset. Table S5: Model hyperparameters across different variable sets. Table S6: Comparison of confusion matrices trained with and without SMOTE. Figure S1: Detailed workflow of machine learning model development. Figure S2: Statistics of missing variables for each patient. Figure S3: Percentage of missing values across clinical variables. Figure S4: Calibration curves with corresponding Brier scores for the final RF and streamlined MLP models on the independent validation dataset. Figure S5: SHAP waterfall plots for representative patients across different predicted risk groups.
