# Supplementary material for: Optimizing In-Hospital Mortality Prediction After Cardiac Surgery: A Machine Learning Approach Using Feature Engineering for Imbalanced Data
Source: Diagnostics (Basel). 2026 Aug 13;16(16):2557. doi: 10.3390/diagnostics16162557 (PMC13512290; doi:10.3390/diagnostics16162557)
Supplement: Supplementary file 1 [file diagnostics-16-02557-s001.zip › diagnostics-4441545-supplementary.pdf]

Supplementary Table S1. Hyperparameter optimization settings and search spaces

Model	Hyperparameter	Search space (68 variables)	Search space (17 variables)
**LR**	C	$10^{-4} – 10^{4}$	$10^{-4} – 10^{4}$
**RF**	Number of trees (*n_estimators*)	100 – 1000 (step = 50)	500–1000 (step = 50)
	Maximum tree depth (*max_depth*)	2 – 18 (step = 2)	5, 10, 20, 30, None
	Maximum features (*max_features*)	sqrt, log2	sqrt, log2, 0.1, 0.3, 0.5, 0.7
	Minimum samples split (*min_samples_split*)	2, 4, 6, 8, 10	2, 5, 10, 20
	Minimum samples leaf (*min_samples_leaf*)	1, 2, 3, 4	1, 2, 3, 4, 8, 16
**XGBoost**	Number of trees (*n_estimators*)	100 – 800 (step = 50)	100 – 800 (step = 50)
	Learning rate	0.01 – 1.0 (step = 0.01)	0.01 – 1.0 (step = 0.01)
	Subsample	0.5, 0.6, 0.7, 0.8, 0.9, 1.0	0.5, 0.6, 0.7, 0.8, 0.9, 1.0
	Column sample by tree (*colsample_bytree*)	0.5, 0.6, 0.7, 0.8, 0.9, 1.0	0.5, 0.6, 0.7, 0.8, 0.9, 1.0
	Maximum tree depth (*max_depth*)	2 – 15	2 – 15
	Minimum child weight (*min_child_weight*)	1 – 8	1 – 8
**MLP**	Hidden layer sizes	(100,100), (32,16,8), (64,32,16), (128,64,32), (64,64,32), (16,8,4)	(100,), (100,100), (50,50,50), (30,30,30), (50,50), (30,30), (50,30,10), (10,10,5)
	Activation	relu, tanh, logistic	relu, tanh, logistic
	Alpha	0.0001, 0.001, 0.01	0.0001, 0.001, 0.01
	Learning rate	constant, adaptive	constant, adaptive

Note: Hyperparameter optimization was performed using RandomizedSearchCV with 10-fold stratified cross-validation. The optimal hyperparameters were selected according to the mean recall score. For each model, up to 200 hyperparameter combinations were randomly evaluated. If the predefined search space contained fewer than 200 unique combinations, all possible combinations were evaluated.

Supplementary Figure S1. Detailed workflow of machine learning model development

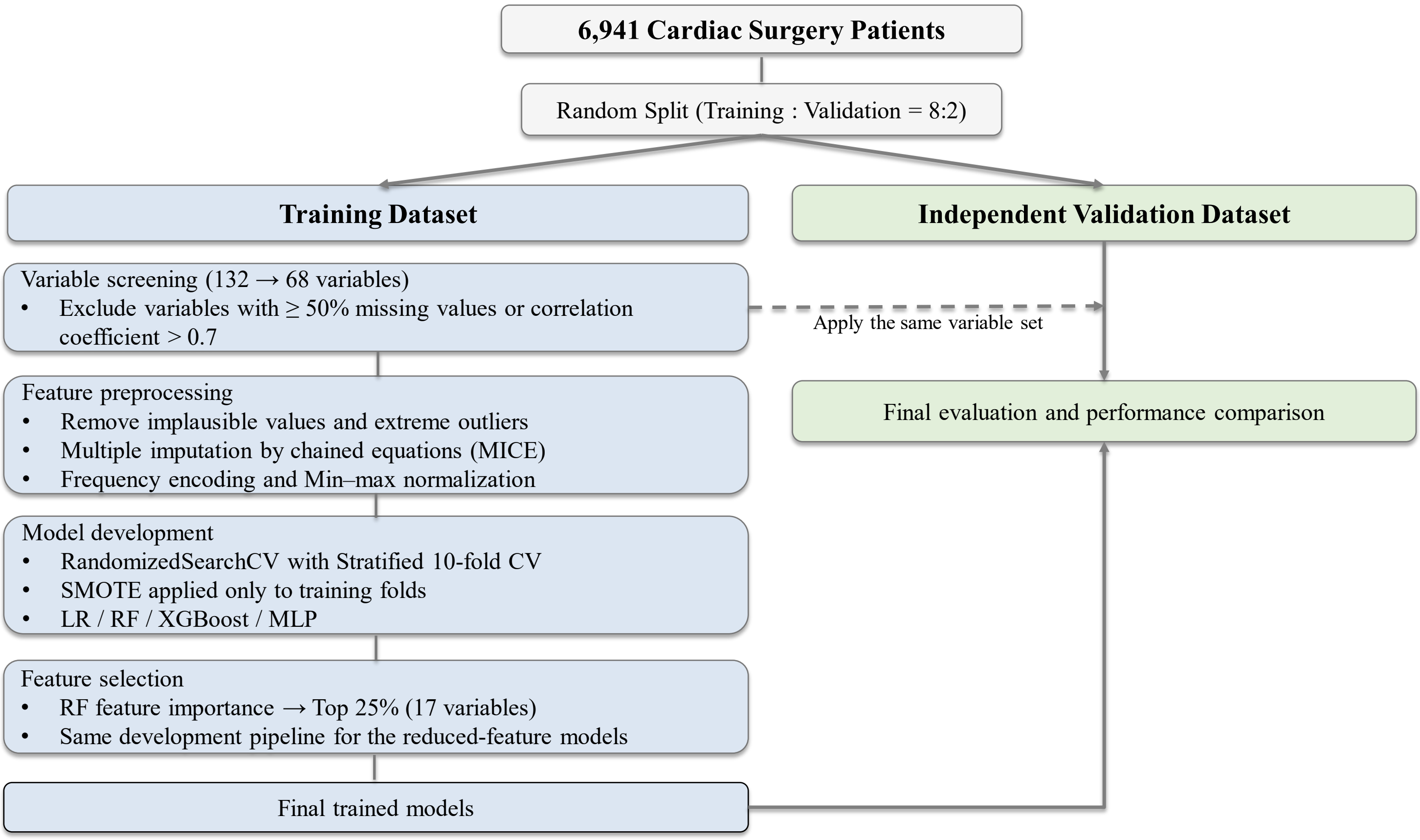

Supplementary Table S2. Distribution of cardiac surgery types by number of cases

**Surgical procedure**	**Cases**	**Percentage (n = 6,941)**
CABG	3771	54.3%
AV	1227	17.7%
MV	540	7.8%
TV	33	0.5%
PV	1	0.0%
CABG + AV	824	11.9%
CABG + MV	218	3.1%
CABG + TV	17	0.2%
AV + MV	106	1.5%
AV + TV	17	0.2%
MV + TV	78	1.1%
CABG + AV + MV	51	0.7%
CABG + AV + TV	5	0.1%
CABG + MV + TV	26	0.4%
AV + MV + TV	21	0.3%
CABG + AV + MV + TV	6	0.1%

Abbreviations: CABG, coronary artery bypass grafting; AV, aortic valve–related procedures; MV, mitral valve–related procedures; TV, tricuspid valve–related procedures; PV, pulmonary valve–related procedures.

Supplementary Figure S2. Statistics of missing variables for each patient

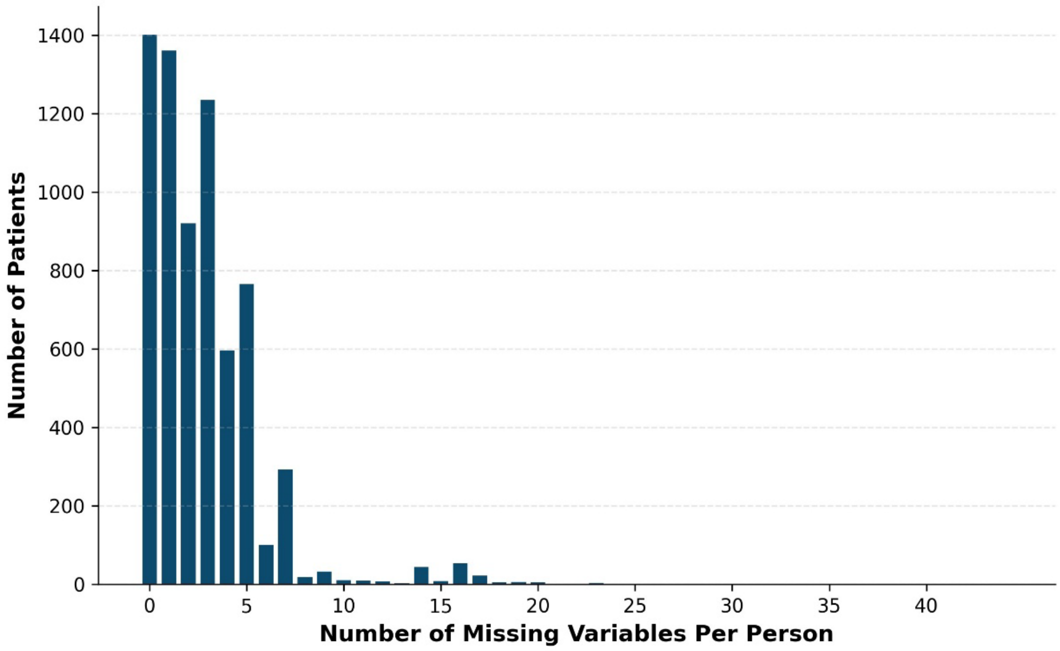

Supplementary Figure S3. Percentage of missing values across clinical variables

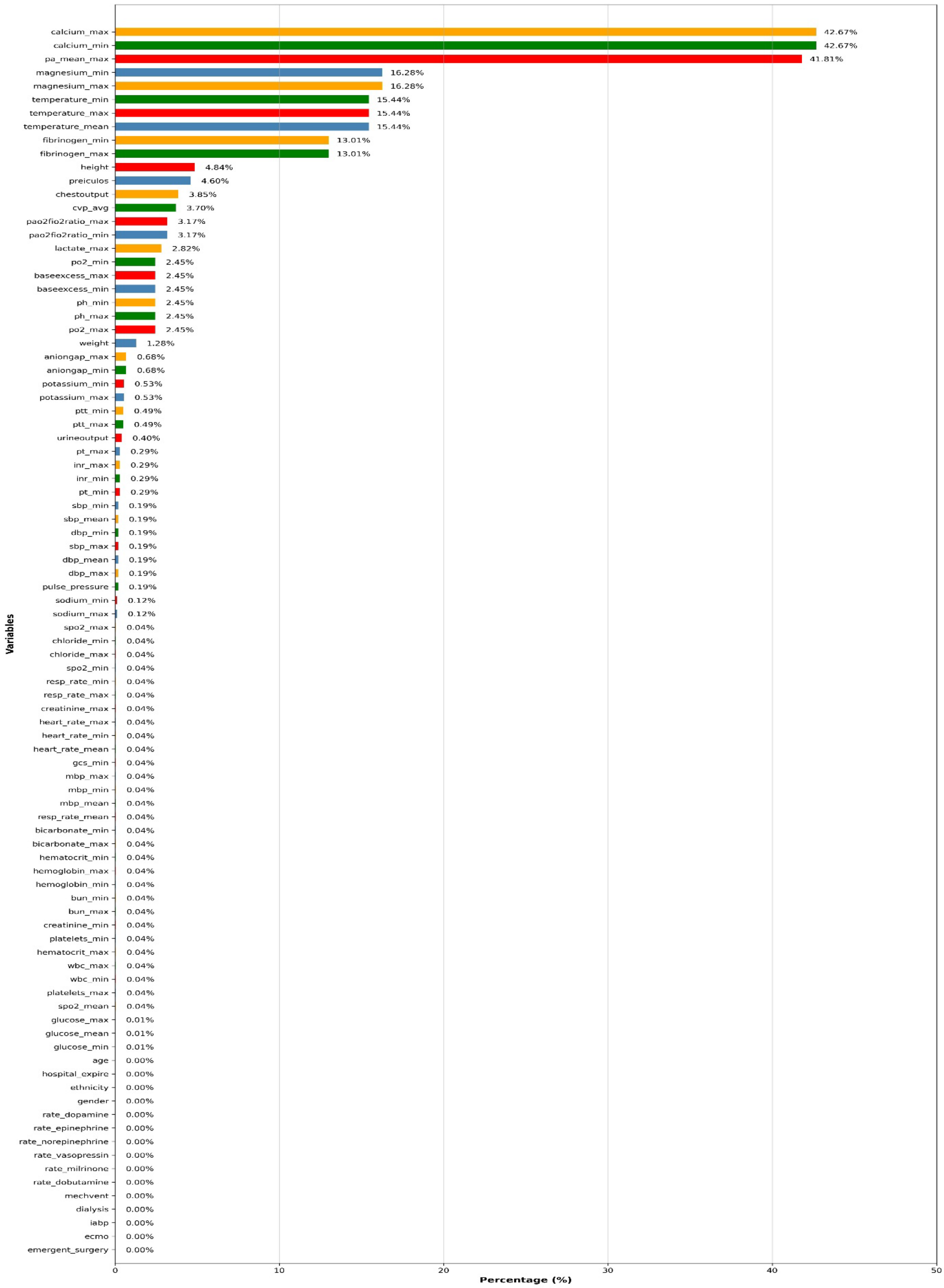

Supplementary Table S3. Descriptive statistics for all variables

	**Survivors (*n* =5491)**	**Nonsurvivors *(n* = 61)**	***p*-value**
**Clinical characteristics**			
Sex			
Women	1560 (28.1)	11 (0.2)	0.15
Men	3931 (70.8)	50 (0.9)	
Ethnicity			
White	4099 (73.8)	40 (0.7)	0.15
Black	200 (3.6)	4 (0.1)	
Latino	162 (2.9)	1 (0)	
Asian	115 (2.1)	2 (0)	
American Native	7 (0.1)	0 (0)	
Others	280 (5.0)	1 (0)	
Unknown	628 (11.3)	13 (0.2)	
Age	68.2 ± 11.3	70.4 ± 12.5	0.12
Height, cm	171.0 ± 10.1	169.3 ± 11.7	0.22
Weight, kg	85.2 ± 19.2	86.2 ± 20.4	0.69
**Monitor values**			
Heart rate, /min			
minimal	67.4 ± 9.8	69.8 ± 14.3	0.19
maximal	97.1 ± 13.2	101.6 ± 16.5	0.01
Systolic blood pressure, mmHg			
minimal	85.9 ± 10.5	77.5 ± 15.1	< 0.001
maximal	141.1 ± 15.5	141.2 ± 27.5	0.97
mean	111.8 ± 7.7	106.4 ± 10.8	< 0.001
Diastolic blood pressure, mmHg			
minimal	44.1 ± 6.8	40.4 ± 9.7	0.01
maximal	75.5 ± 12.6	75.8 ± 17.1	0.90
Respiratory rate, /min			
minimal	10.5 ± 3.0	10.7 ± 4.2	0.76
maximal	26.8 ± 5.4	27.6 ± 6.4	0.24
mean	17.7 ± 2.6	18.9 ± 3.4	0.01
Temperature, ℃			
minimal	35.9 ± 0.7	35.7 ± 0.9	0.07
maximal	37.3 ± 0.5	37.3 ± 0.7	0.95
mean	36.7 ± 0.4	36.6 ± 0.5	0.32
SpO_2, %			
minimal	92.9 ± 4.3	89.9 ± 11.6	0.045

maximal	99.9 ± 0.3	99.7 ± 0.9	0.07
mean	97.8 ± 1.4	97.2 ± 4.0	0.21
Pulmonary artery pressure, mmHg			
Mean	30.9 ± 7.7	41.4 ± 12.3	< 0.001
Central venous pressure, mmHg			
Mean	10.8 ± 4.9	15.3 ± 7.1	< 0.001
**Laboratory values**			
Glucose, mg/dL			
minimal	87.2 ± 15.8	91.2 ± 28.8	0.29
maximal	187.1 ± 38.7	239.4 ± 91.9	< 0.001
White blood cells, $\times 10^3$/uL			
minimal	10.4 ± 4.1	10.1 ± 4.9	0.59
maximal	16.6 ± 6.3	17.5 ± 7.4	0.23
Hemoglobin, g/dL			
minimal	9.1 ± 1.5	8.1 ± 1.7	< 0.001
maximal	11.3 ± 1.5	11.3 ± 2.0	0.84
Platelets, $\times 10^3$/uL			
minimal	137.3 ± 50.5	123.8 ± 72.8	0.16
Fibrinogen, mg/dL			
minimal	215.5 ± 71.4	187.7 ± 88.4	0.02
International normalized ratio (INR)			
minimal	1.2 ± 2.0	1.3 ± 0.4	0.06
maximal	1.5 ± 0.4	2.1 ± 1.9	0.01
Activated partial thromboplastin time (aPTT), sec			
minimal	29.1 ± 5.4	33.8 ± 9.9	< 0.001
maximal	40.0 ± 20.1	67.8 ± 38.4	< 0.001
Blood urea nitrogen (BUN), mg/dL			
minimal	16.9 ± 9.5	28.8 ± 18.5	< 0.001
Creatinine, mg/dL			
minimal	1.0 ± 0.8	2.1 ± 2.1	< 0.001
Sodium, mEq/L			
Minimal	137.1 ± 2.8	137.1 ± 4.3	0.98
Maximal	139.2 ± 2.9	140.8 ± 4.6	0.01
Potassium, mEq/L			
Minimal	4.2 ± 0.5	4.1 ± 0.7	0.77

Maximal	4.7 ± 0.5	5.0 ± 0.8	0.004
Calcium, mg/dL			
minimal	8.3 ± 0.6	8.4 ± 0.7	0.20
maximal	8.4 ± 0.6	8.9 ± 0.9	< 0.001
Magnesium, mg/dL			
minimal	2.3 ± 0.4	2.3 ± 0.5	0.62
maximal	2.6 ± 0.5	2.8 ± 0.7	< 0.001
Chloride, mEq/L			
minimal	104.7 ± 3.9	102.6 ± 6.4	0.01
maximal	109.0 ± 3.8	107.7 ± 6.1	0.11
Bicarbonate, mEq/L			
minimal	22.4 ± 2.3	19.5 ± 3.3	< 0.001
Anion gap, mEq/L			
minimal	10.7 ± 2.4	13.7 ± 3.7	< 0.001
maximal	12.9 ± 2.8	18.0 ± 5.5	< 0.001
Lactate, mmol/L			
maximal	2.8 ± 1.3	6.2 ± 4.1	< 0.001
pH			
minimal	7.31 ± 0.05	7.24 ± 0.13	< 0.001
maximal	7.45 ± 0.05	7.45 ± 0.06	0.74
PaO_2, mmHg			
minimal	107.0 ± 33.9	95.3 ± 39.7	0.01
maximal	400.9 ± 76.4	387.3 ± 140.2	0.45
P/F ratio			
maximal	351.7 ± 118.1	366.2 ± 151.8	0.46
Base excess, mmol/L			
minimal	-3.1 ± 2.6	-7.1 ± 6.0	< 0.001
maximal	2.1 ± 2.3	1.3 ± 3.2	0.09
**Clinical records**			
Emergent Surgery	4250 (76.5)	43 (0.8)	< 0.001
Mechanical Ventilation	3396 (61.2)	50 (0.9)	< 0.001
ECMO	0 (0)	3 (0.1)	0.08
IABP	153 (2.8)	13 (0.2)	< 0.001
Dialysis	78 (1.4)	10 (0.2)	< 0.001
Minimal GCS	13.1 ± 3.77	12.902 ± 4.241	0.65
Pre-ICU length of stay, hr	52.2 ± 77.2	110.5 ± 196.7	0.02
Maximal VIS	3.0 ± 8.9	26.7 ± 30.9	< 0.001
Urine output, ml	1972.5 ± 927.3	1250.4 ± 1247.1	< 0.001
Chest tube output, ml	654.01 ± 379.6	1431.9 ± 1390.1	< 0.001

Supplementary Table S4. Model performance on the training dataset

Model	Accuracy	Precision	Recall	F1-score	ROC/AUC
**LR**	0.834 +/- 0.020	0.040 +/- 0.014	0.610 +/- 0.216	0.075 +/- 0.026	0.778 +/- 0.125
**RF**	0.901 +/- 0.009	**0.069 +/- 0.029**	**0.640 +/- 0.274**	**0.125 +/- 0.053**	**0.878 +/- 0.091**
**XGBoost**	0.859 +/- 0.008	0.045 +/- 0.021	0.593 +/- 0.284	0.083 +/- 0.039	0.822 +/- 0.122
**MLP**	**0.928 +/- 0.016**	0.056 +/- 0.036	0.310 +/- 0.150	0.094 +/- 0.056	0.692 +/- 0.103

Abbreviations: LR, Logistic Regression; RF, Random Forest; XGBoost, eXtreme Gradient Boosting; MLP, Multi-Layer Perceptron; ROC/AUC, Receiver Operating Characteristic Area Under the Curve.

Supplementary Table S5. Model hyperparameters across different variable sets

Model	Hyperparameter	Selected Hyperparameters
LR	C	All variables: 0.001<br>17 variables: 0.1
RF	n_estimators	All variables: 650<br>17 variables: 1000
	max_depth	All variables: 2<br>17 variables: 2
	min_samples_split	All variables: 8<br>17 variables: 3
	min_samples_leaf	All variables: 2<br>17 variables: 3
	max_features	All variables: “sqrt”<br>17 variables: “log2”
XGboost	n_estimators	All variables: 100<br>17 variables: 100
	max_depth	All variables: 2<br>17 variables: 2
	subsample	All variables: 0.6<br>17 variables: 0.6
	colsample_bytree	All variables: 0.8<br>17 variables: 0.8
	learning_rate	All variables: 0.01<br>17 variables: 0.01
	min_child_weight	All variables: 3<br>17 variables: 3
MLP	hidden_layer_sizes	All variables: (30,10,5)<br>17 variables: (100, 100)
	max_iter	All variables: 2000<br>17 variables: 2000
	activation	All variables: “logistic”<br>17 variables: “logistic”
	alpha	All variables: 0.01<br>17 variables: 0.0001
	learning_rate	All variables: “constant”<br>17 variables: “constant”

Supplementary Table S6. Comparison of confusion matrices trained with and without SMOTE

Logistic Regression

Without SMOTE

		Truth	
		death	alive
Predict	death	2	3
	alive	13	1371

Recall: 0.133

With SMOTE

		Truth	
		death	alive
Predict	death	8	219
	alive	7	1155

Recall: 0.533

Random Forest

Before SMOTE

		Truth	
		death	alive
Predict	death	2	2
	alive	13	1372

Recall: 0.133

After SMOTE

		Truth	
		death	alive
Predict	death	11	169
	alive	4	1205

Recall: 0.733

XGBoost

Before SMOTE

		Truth	
		death	alive
Predict	death	1	11
	alive	14	1363

Recall: 0.067

After SMOTE

		Truth	
		death	alive
Predict	death	12	222
	alive	3	1152

Recall: 0.8

Multilayer Perceptron

Before SMOTE

		Truth	
		death	alive
Predict	death	3	2
	alive	12	1372

Recall: 0.2

After SMOTE

		Truth	
		death	alive
Predict	death	7	97
	alive	8	1277

Recall: 0.467

Note: Performance and confusion matrices of models trained without and with SMOTE, evaluated on the independent validation dataset.

Supplementary Figure S4. Calibration curves with corresponding Brier scores for the final RF and streamlined MLP models on the independent validation dataset

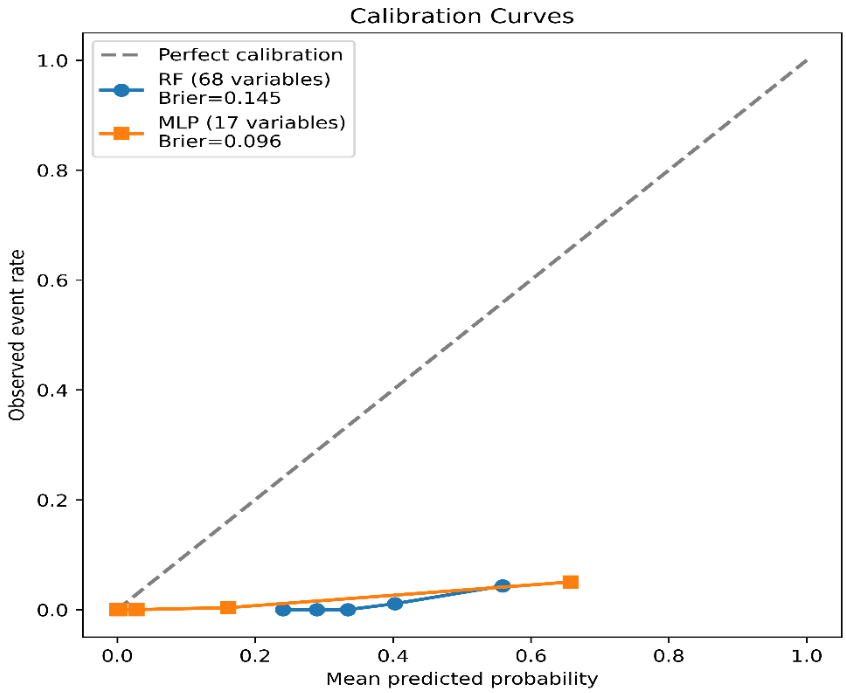

Supplementary Figure S5. SHAP waterfall plots for representative patients across different predicted risk groups

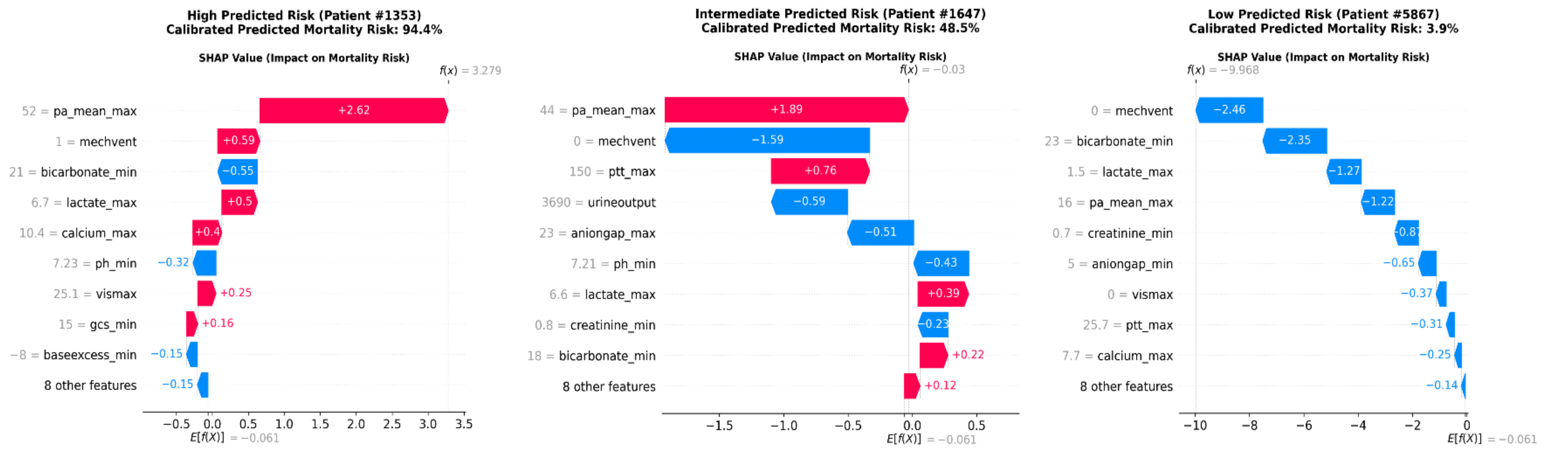

Note: Representative SHAP waterfall plots are shown for patients with high-, intermediate-, and low-predicted mortality risk. SHAP values are presented in the log-odds space of the original MLP model to preserve the additive property of SHAP. The predicted probabilities displayed above each panel were obtained using post-hoc Platt scaling for visualization purposes only, thereby providing clinically interpretable individual risk estimates. Accordingly, the displayed probabilities do not directly correspond to the SHAP values shown in the waterfall plots.
